# Supplementary material for: Impact of Experimental Conditions on Extracellular Vesicles’ Proteome: A Comparative Study
Source: Life (Basel). 2023 Jan 11;13(1):206. doi: 10.3390/life13010206 (PMC9864048; doi:10.3390/life13010206)
Supplement: Supplementary file 1 [file life-13-00206-s001.zip › Supplementary Figures.pdf]

life

MDPI

Supplementary Materials

Impact of Experimental Conditions on Extracellular Vesicles' Proteome: A Comparative Study

Tímea Böröczky[1,2,3], Gabriella Dobra[1,2,3], Mátyás Bukva[1,2,3], Edina Gyukity-Sebestyén[1,2], Éva Hunyadi-Gulyás[4], Zsuzsanna Darula[4,5], Péter Horváth[1,6], Krisztina Buzás[1,2†*] and Mária Harmati[1,2†*]

[1] Laboratory of Microscopic Image Analysis and Machine Learning, Institute of Biochemistry, Biological Research Centre, Eötvös Lorand Research Network (ELKH), H-6726 Szeged, Hungary; boroczky.timea@brc.hu (T.B.); dobra.gabriella@brc.hu (G.D.); bukva.matyas@brc.hu (M.B.); sebestyen.edina@brc.hu (E.G.-S.); horvath.peter@brc.hu (P.H.); buzas.krisztina@brc.hu (K.B.); harmati.maria@brc.hu (M.H.)
[2] Department of Immunology, Albert Szent-Györgyi Medical School, Faculty of Science and Informatics, University of Szeged, H-6720 Szeged, Hungary
[3] Doctoral School of Interdisciplinary Medicine, Albert Szent-Györgyi Medical School, University of Szeged, H-6720 Szeged, Hungary
[4] Laboratory of Proteomics Research, Biological Research Centre, Eötvös Lorand Research Network (ELKH), H-6726 Szeged, Szeged, Hungary; gulyas.eva@brc.hu (E.H.-G.)
[5] Single Cell Omics Advanced Core Facility, Hungarian Centre of Excellence for Molecular Medicine Szeged, Hungary; darula.zsuzsanna@brc.hu (Z.D.)
[6] Institute for Molecular Medicine Finland (FIMM), University of Helsinki, 00290 Helsinki, Finland
[*] Correspondence: harmati.maria@brc.hu; buzas.krisztina@brc.hu; Tel.: +36-62-432-340 (K.B., M.H.)
[†] These authors contributed equally to this work

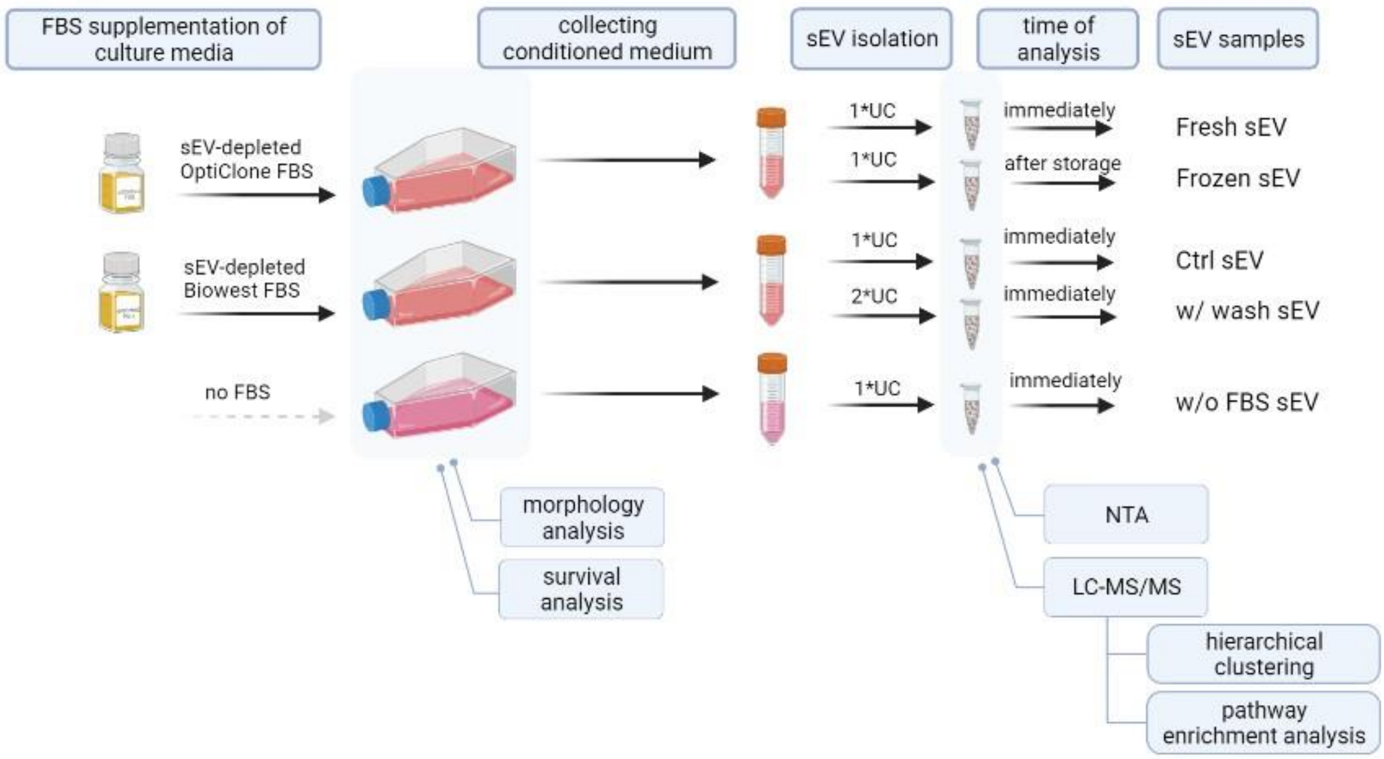

Figure S1. Experimental workflow. B16F1 mouse melanoma cells were cultured in sEV-depleted serum-containing (OptiClone or Biowest FBS) or serum-free medium. For morphology and survival analysis, cells were monitored daily and counted with trypan blue staining after 72 hours. On the third day conditioned media were harvested and vesicles were isolated by filtration and differential ultracentrifugation. Three sample types were measured immediately (Fresh sEV, Ctrl sEV, w/o FBS sEV); the w/ wash sEV sample was generated by an additional washing step, i.e., re-suspension in PBS and a second round of ultracentrifugation; and the Frozen sEV sample was stored at −80 °C for 3 months. Vesicle isolates were analyzed with NTA and LC-MS/MS. Heatmaps were generated by hierarchical clustering using the MORPHEUS program, and pathway enrichment analyses were performed using the ShinyGO 0.76.3 software with the default parameters. Abbreviations: FBS - fetal bovine serum, LC-MS/MS – liquid chromatography - tandem mass spectrometry, NTA – nanoparticle tracking analysis, sEV – small extracellular vesicle, UC – ultracentrifugation. Figure was created with BioRender.com.

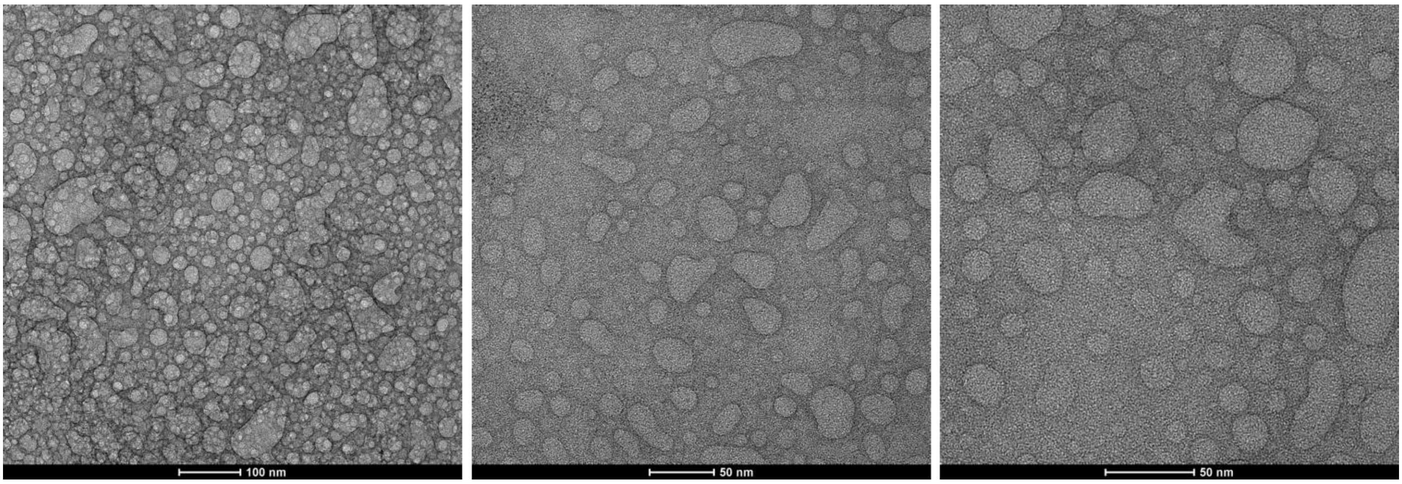

Figure S2. Transmission electron microscopy of the Ctrl sEV isolates. Images show the vesicles in different dilutions and magnifications.

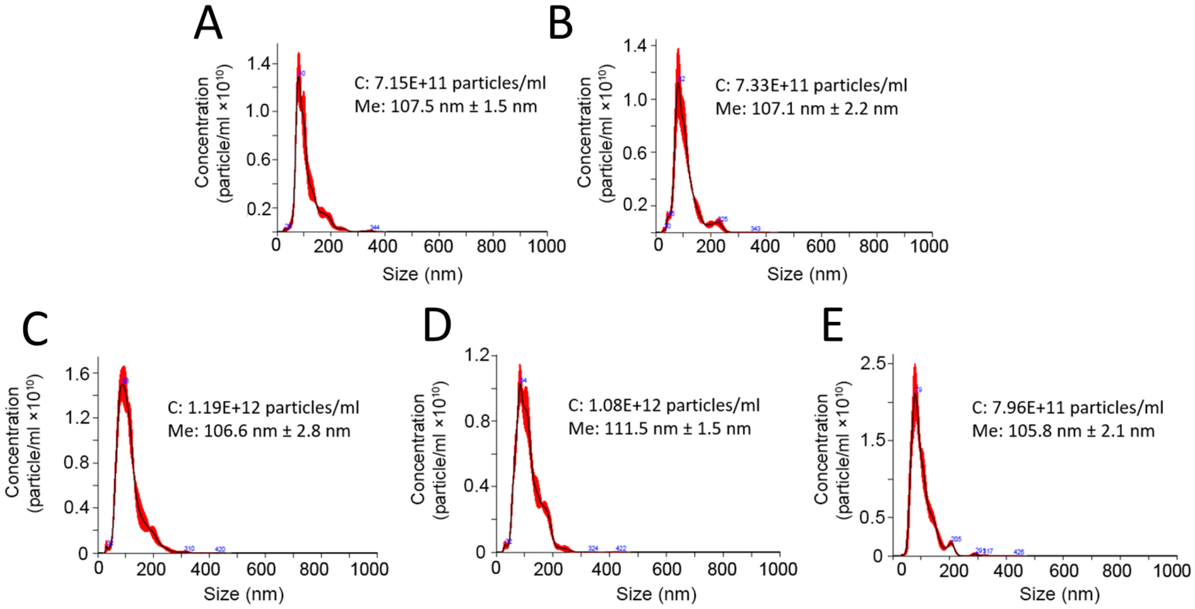

Figure S3. Nanoparticle tracking analysis of the five sEV samples. The figures represent the size distribution of the isolated Fresh sEV **(A)**, Frozen sEV **(B)**, Ctrl sEV **(C)**, w/o FBS sEV **(D)**, and w/ wash sEV **(E)** samples (black and red lines represent the mean and the standard deviation of the concentration; C: particle concentration; Me: mean diameter size).

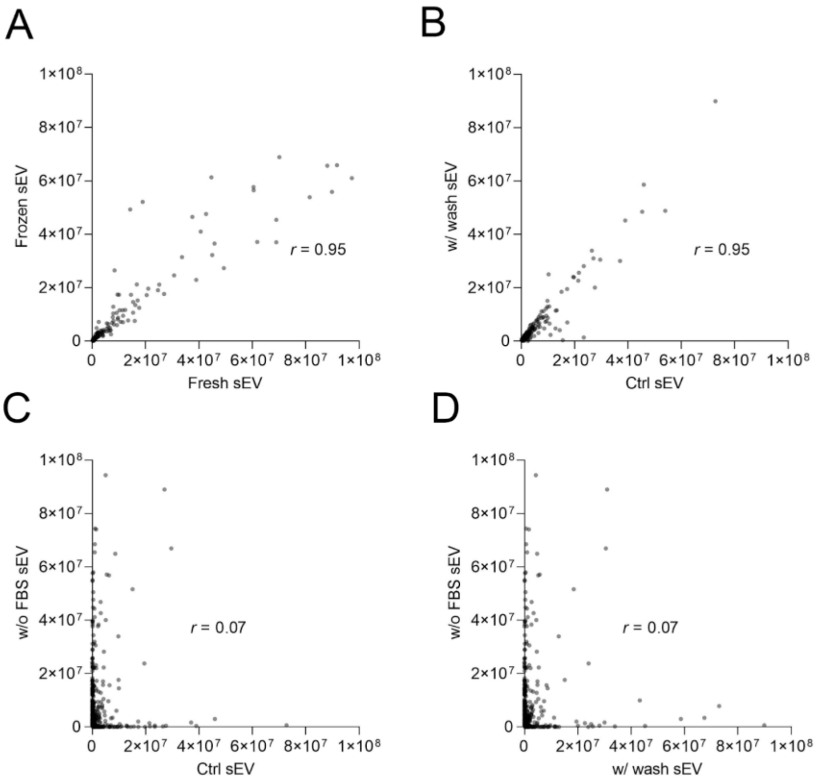

Figure S4. Pearson's correlation analysis on the protein content of the five sEV samples. Dot plots show the relationship between the Fresh sEV and Frozen sEV samples **(A)** and also between the Ctrl sEV and w/ wash sEV **(B)**, Ctrl sEV and w/o FBS sEV **(C)**, and also between the w/ wash sEV and the w/o FBS sEV samples **(D)**. Correlation coefficients were obtained using Pearson's correlation analysis.

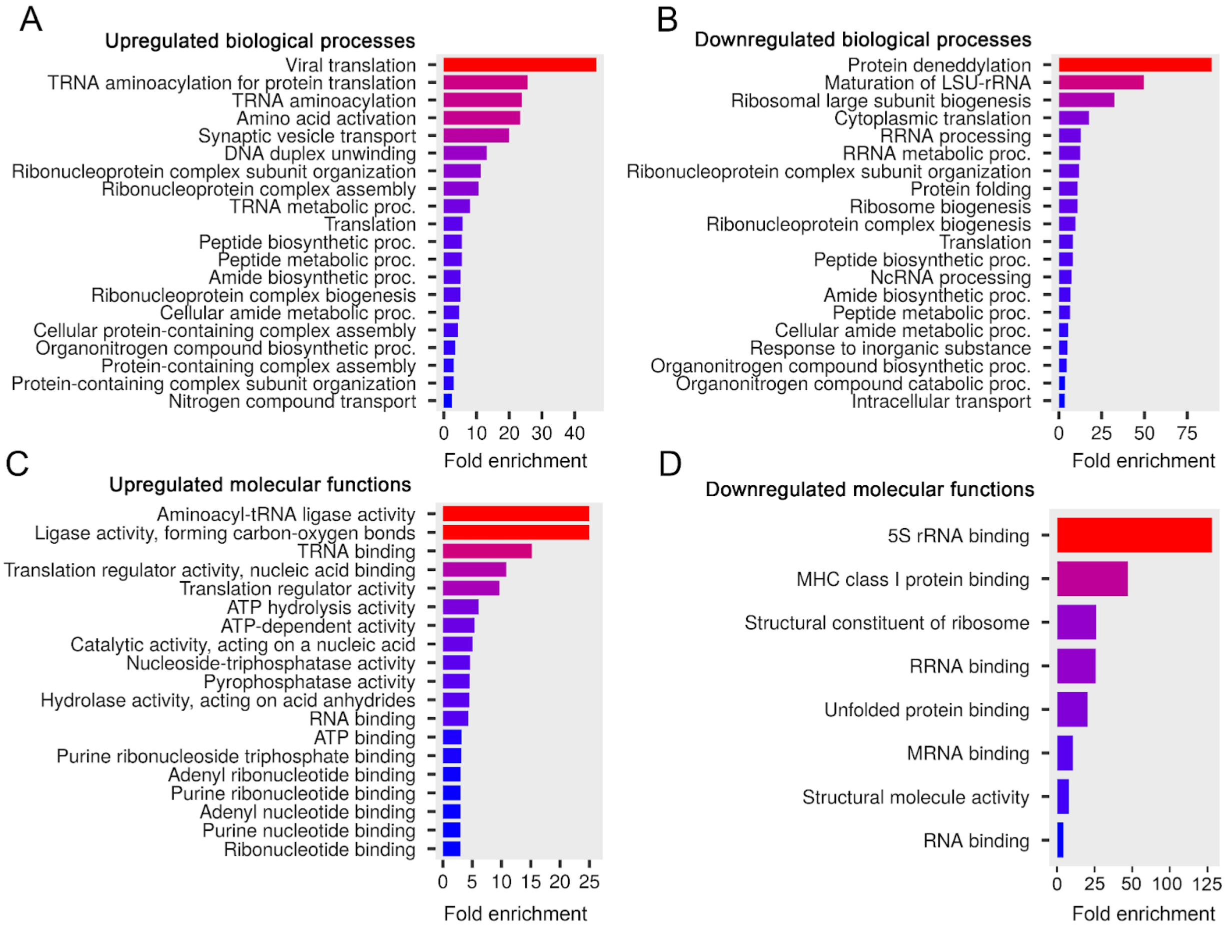

Figure S5. The influence of PBS washing on the biological and molecular functions of the melanoma proteins. Bar charts show the top 20 GO terms for the elevated **(A)** and downregulated **(B)** biological processes—ranked by fold enrichment—of the associated mouse proteins in the w/ wash sEV sample as compared to the Ctrl sEV sample. Bar charts show the top 20 GO terms for the elevated **(C)** and downregulated **(D)** molecular functions—ranked by fold enrichment—of the associated mouse proteins in the w/ wash sEV sample as compared to the Ctrl sEV sample. Changing colors of the bars represent the decreasing fold enrichment values.
